# Association of the *CYP1A1* rs4646903 polymorphism with susceptibility and severity of coronary artery disease 

**DOI:** 10.22099/mbrc.2021.39141.1574

**Published:** 2021-06

**Authors:** Mahsa Eskandari, Ali Awsat Mellati, Khalil Mahmoodi, Koorosh Kamali, Mohammad Soleiman Soltanpour

**Affiliations:** 1 Zanjan Metabolic Diseases Research Center, Zanjan University of Medical Sciences, Zanjan, Iran; 2 Department of Cardiology, School of Medicine, Zanjan University of Medical Sciences, Zanjan, Iran; 3 Social Determinants of Health Research Center, Zanjan University of Medical Sciences, Zanjan, Iran; 4 Department of Medical Laboratory Sciences, School of Allied Medical Sciences, Zanjan University of Medical Sciences, Zanjan, Iran

**Keywords:** Coronary artery disease, CYP1A1, rs4646903, Polymorphism, PCR-RFLP

## Abstract

Given the significant physical, mental, and economic problems of coronary artery disease (CAD), it is important for communities to help reduce these costs. The Cytochrome P450 Family 1 Subfamily A Member 1) CYP1A1 (enzyme is known to cause coronary artery disease through various mechanisms. Therefore, it is important to investigate the polymorphisms that affect the activity of this enzyme. After collecting samples from 191 patients with angiographically verified CAD and 191 healthy individuals, genotyping for *CYP1A1* rs4646903 polymorphism was carried out. Lipid profile was assessed by conventional colorimetric method. The results showed that the frequency of heterozygous and homozygous mutant genotypes of rs4646903 polymorphism was 36.6% and 5.2% in patients and 20.9% and 2.1% in controls, respectively. The heterozygous genotype (OR=2.24; 95% CI=1.30-3.84, P=0.003), homozygous mutant genotype (OR=3.97; 95% CI=1.05-14.98, P=0.042) and mutant C allele (OR=2.15; 95% CI=1.46-3.15, P<0.001) was significantly associated with CAD risk. Further analysis identified *CYP1A1* rs4646903 polymorphism as a significant risk factor for early onset (P= 0.005) but not late onset (P=0.066) CAD. However, the frequency of heterozygous and homozygous mutant genotype of rs4646903 polymorphism did not differ significantly among the CAD patients with various number of stenotic vessel (P>0.05). In conclusion, the rs4646903 polymorphism contributed to the susceptibleness of people to CAD.

## INTRODUCTION

Coronary artery disease (CAD) or ischemic heart disease occurs when heart arteries disable to supply oxygenated blood to the heart muscle especially because of plaque formation in the intima tunica of heart arteries [1]. In 2017 based on the global burden of disease research 15.96 and 25.15 percent of total deaths, respectively, in the world and Iran are due to coronary heart disease [2]. This figure is forecasted to reach in order 13.35% and 20.71% up to 2040 [3]. Several environmental agents and multi-gene are involved in CAD. It is substantial to identify genetic risk factors correlated to CAD, screen them in high-risk individuals, and adopt methods to reduce the complications of these risk factors.

CYP1A1, a heme-thiolate protein [4], is a momentous phase I enzyme [5]. The *CYP1A1* gene variants was reported as a risk factor for different disease such as cancer [6]. Some other studies reported that the genetic polymorphisms of *CYP1A1* gene contributes to the pathogenesis of CAD [7]. CYP1A1 pertain to the cytochrome P450 family that placed on 15q24.1 [5]. Polycyclic aromatic hydrocarbons (PAH) metabolic intermediates result from this enzyme activity and 2,3,7,8-tetrachlorodibenzo-p-dioxin (TCDD) by the induction of CYP1A1 play a consequential role in the development of atherosclerotic lesions, hypertension, and decreased cholesterol reverse transportation [8-12].

PAH and TCDD are two xenobiotic substances. They are by-products of combustion include the burning of fossil fuels, waste and industrial processes, but may also occur as a result of natural processes such as volcanic eruptions or forest fires [13-15]. In people who are not in highly polluted environments (not working in the production of PAHs or related materials) and non-smokers, food is a major source of PAHs. In smokers, respectively, the most important exposure ways are smoking and food consumption [16]. As some cigarette smoke enters the air where others breathe, others are also exposed to all diseases caused by cigarette smoke [17]. TCDD accumulates in the food chain. Human exposure mainly occurs through the consumption of contaminated food [18].

 Single nucleotide polymorphisms that alter *CYP1A1* expression or function are expected to influence the susceptibleness to cardiovascular disease. The rs4646903 mutation of the *CYP1A1* gene (T>C) that happen at the downstream polyadenylation site at the 3'UTR non-coding section leads to an increase in the half-life of the mRNA, which is characterized by an increase in the concentration and enzymatic activity of CYP1A1 [19, 20], which in turn can increase the CYP1A1 metabolite level and increased risk of heart disease. 

Since reported results are conflicting [7, 21-23] and no studies have been conducted in Iran regarding the correlation of T6235C polymorphism of *CYP1A1* gene with CAD, investigation of this polymorphism in Iranian patients with CAD seems necessary and may elucidate the pathological process of CAD and reduces the side effects of non-specific therapies.

## MATERIALS AND METHODS

**Subject:** 191 CAD patients and 191control individuals selected under the supervision of Ayatollah Mousavi teaching hospital cardiologist in Zanjan, Iran. The inclusion and exclusion criteria for CAD patients and control subjects were according to our previously published article [24]. Data on hyperlipidemia, smoking habit, high blood pressure (specified as systolic blood pressure >140 mmHg and/or diastolic blood pressure >90 mmHg) and diabetes (specified as fasting blood glucose> 126 mg/dl) were gathered. Informed consent was acquired from whole of the subjects. Ethics Committee of Zanjan University of Medical Sciences (Code of Ethics: ZUMS.REC. 1398.432) confirmed this research.

Blood drawing and processing: Intravenous blood samplings were done after 12 hours of fasting. The blood samples were then transferred to a tube containing ethylenediaminetetraacetic acid (EDTA) and centrifuged. Plasma sections were used for biochemical measurements and cell sections were used for DNA isolation. Then plasmas were stored at -20°C until testing.

**Biochemical assays** **:** Lipid panel (high-density lipoprotein cholesterol (HDL-C), low-density lipoprotein cholesterol (LDL-C), triglycerides (TG), cholesterol, and blood glucose level of the samples were measured by calorimetric method (Pars Azmoon Co., Tehran, Iran). 

**Genotyping** **:** DNA of leukocytes was isolated using a trading DNA isolation kit (Yekta Tajhiz Azma, Tehran, Iran) according to the instructions of its company. A 373-bp fragment including the diversity site was amplified with Polymerase Chain Reaction (PCR) using a standard PCR protocol as previously described [24]. The sequence of primers were as follows, F: 5ʹAGC AGT GAA GAG GTG TAG CCG‐3ʹ and R: 5ʹTAG AGA GGG CGT AAG TCA GCA‐3ʹ as defined previously [25]. For RFLP analysis, the amplified DNA fragment was subjected to 0.5 units of *Msp*I (EURx., Gdansk, Poland ) restriction enzyme at 37° C for one hour. The broken-down PCR products were electrophoresed on a 2.5% agarose gel. At the presence of the C allele, a cleavage site for *MspI* enzyme is created and restriction digestion produces two unequal 138 bp and 235bp bands. The genotyping results of some samples were confirmed by the Sanger sequencing method (Supplement Fig. 1).

**Statistical analysis:** In this case-control study, the numerical information was reported as mean±SD and was contrasted using the Student t-test. The variables reported as absolute and percentage that using the Chi-square test or Fisher's exact tests were contrasted. Logistic regression analysis was used to detect the independent relation of each risk factor with CAD. The statistical analysis was carried out by SPSS 16 (SPSS Inc., Chicago, USA) software. P-values lower than 0.05 was considered significant.

## RESULTS

In the present study 191 patients with CAD and 191 healthy individuals confirmed by a cardiologist, made up our statistical population. The mean age of CAD patients and control group were 58.95±11.53 and 57.68±16.25, respectively. 95 CAD patients and 93 control subjects were male. Some medical data of individuals including the TG (P<0.001), cholesterol (P<0.001) and LDL-C (P<0.001) levels, smoking (P=0.002), diabetes (P=0.001) and high-blood pressure (P<0.001) were significantly related to CAD risk. However, no significant association was found between age (P= 0.382), sex (P= 0.838), HDL-C levels (P= 0.926) and risk of CAD (Supplementary Table 1). 

The results showed that the frequency of heterozygous and mutant homozygous genotypes of rs4646903 polymorphism was 36.6% and 5.2% in patients and 20.9% and 2.1% in controls, respectively. The diversity of rs4646903 genotypes was in accordance with Hardy-Weinberg principle in both CAD group (P=0.808) and control group (P=0.516). The chance of developing CAD in subjects with heterozygous genotype was about 2.31 times of control group (OR=2.31; 95% CI=1.46-3.66, P<0.001). Also, the mutant homozygous genotype (CC) significantly elevated the risk of CAD (OR=3.31; 95% CI=1.08-9.76, P= 0.037). Also, the minor C allele was associated with higher risk of CAD (OR =2.15; 95%CI=1.46-3.15, P<0.001) (Table 1). 

**Table 1:** Genotype and alleles frequency of *CYP1A1* rs4646903 SNP in control and patient group

**Study group**	**Case group n=191**	**Control group n=191**	**P**	**OR (95% CI)**
**TT**	111 (58.1%)	147 (77.0%)	**Reference genotype**	
**TC**	70 (36.6%)	40 (20.9%)	<0.001	2.31 (1.46-3.66)
**CC**	10 (5.2%)	4 (2.1%)	0.037	3.31 (1.08-9.76)

P values were computed by Fisher's exact test.

For assessing the role of *CYP1A1* rs4646903 polymorphism in the development of early onset CAD and/or late onset CAD, the genotypes distribution of rs4646903 polymorphism was compared between subjects under 60 years age and over 60 years age. Results indicated significant variation in the genotypes diversity of rs4646903 polymorphism between case and control group only in the age category under 60 years old (𝜒^2^=10.66, df=2, P=0.005) but not in the age category over 60 years old (𝜒^2^=5.42, df=2, P=0.066) (Table 2). 

**Table 2:** The genotypes distribution of *CYP1A1* rs4646903 polymorphism based on age group

**Genotypes**	**Age group of study population**						
	**<60 years**			**>60 years**			
	**Case**	**Control**		**Case**	**Control**		
**TT**	60 (60.0%)	84 (80.8%)		51 (56.0%)	63 (72.4%)		
**TC**	35 (35.0%)	18 (17.3%)		35 (38.5%)	22 (25.3%)		
**CC**	05 (5%)	02 (1.9%)		05 (5.5%)	2 (2.3%)		

For investigating the independent correlation of each risk agents in CAD pathogenesis, logistic regression analysis was done. Results indicated that the total cholesterol (P<0.001), triglycerides (P=0.011), LDL‑C (P=0.028), diabetes (P=0.024), hypertension (P<0.001), smoking (P=0.003), mutant homozygote genotype (P=0.042) and heterozygote genotype (P=0.003) were independent risk agents of CAD (Table 3). 

**Table 3:** Regression analysis of relation between risk agents and CAD

**Variables**	**Wald**	**OR**	**95% CI**	**P value**
TC vs. TT	8.54	2.236	1.30-3.84	0.003
CC vs. TT	4.13	3.966	1.05-14.98	0.042
High-blood pressure	18.97	4.220	2.21-8.07	<0.001
Diabetes	5.07	2.125	1.10-4.09	0.024
Smoking	9.08	2.589	1.39-4.80	0.003
Triglyceride	6.42	1.004	1.00-1.01	0.011
Cholesterol	16.55	1.010	1.00-1.01	<0.001
HDL-C	0.86	1.010	0.99-1.03	0.355
LDL-C	4.82	1.008	1.00-1.01	0.028
Age	0.03	0.998	0.98-1.01	0.853
Sex	0.11	0.923	0.58-1.46	0.734

Moreover, we surveyed the relationship between genotypes of *CYP1A1* rs4646903 polymorphism and the number of stenotic vessel (1, 2, 3 or more). There was no significant relation between the number of stenotic vessel and the prevalence of heterozygote and mutant homozygote genotypes (P>0.05) (Table 4).

**Table 4:** The number of blocked vessels in CAD patient based on genotypes of *CYP1A1* rs4646903 polymorphism

**Number of stenotic vessel**	**1SV** **n=65**	**2SV** **n=85**	**3SV** **n=41**	**P vale** **(2SV vs.** **1SV)**	**P vale ** **(3SV vs.** **1SV)**
**TT**	36 (55.3%)	55 (64.7%)	20 (48.8%)	Ref	Ref
**TC**	26 (40.0%)	28 (32.9%)	16 (39.0%)	0.385	0.835
**CC**	3 (4.6%)	2 (2.3%)	5 (12.2%)	0.393	0.245

## DISCUSSION

The goal of this case-control study was evaluating the role of rs4646903 polymorphism in development and severity of coronary artery disease in an Iranian subpopulation. The principal outcomes gained from our study were: (I) Heterozygous, homozygous mutant genotypes and mutant C allele of rs4646903 polymorphism elevated CAD risk (II) The *CYP1A1* rs4646903 polymorphism acts as a significant risk factor for development of early onset but not late onset CAD (III) Rs4646903 polymorphism didn’t influence stenosis severity.

As mentioned above xenobiotic metabolic intermediates such as PAH that result from CYP1A1 enzyme activity is consequential for CAD development. In vitro experiments showed that PAH metabolic mediator inhibits liver X receptor (LXR) signal transduction [9], thereby reducing expression of LXR target genes such as *PLTP*, *ABCA1*, *ABCG1*, *ARL7*, *CYP7A1*, *ABCG5*, *ABCG8*, that are contributing to cholesterol pumping-out from the peripheral cell and transport it to the liver to be excreted as bile acid [26]. Metabolic intermediaries of PAH have DNA binding ability [27] and in turn, it causes progression of atherosclerosis [28].

One of the PAH metabolic caused by CYP1A1 activity and TCDD induction of CYP1A1 is the reactive oxygen species (ROS) [8, 10]. Elevated ROS level has a pivotal role in the developing of atherosclerotic lesions and hypertension [11, 29]. Superoxide anions as a ROS react quickly with nitric oxide (NO) and inactivate it [30]. NO is a cardiovascular protective agent [31]. Some polymorphisms of *NO *gene may be associated with decreased plasma NO level [32]. Overexpression of *CYP1A1* in cardiomyocytes reduces mitochondrial membrane quality and elevates mitochondrial ROS [33]. 

Association of rs4646903 polymorphism with CAD has been studied in different countries and different results has been obtained [7, 21-23]. In our study, C allele prevalence of control group was12.6% which was close to the Achour, and et al results (12.2%) [34]. Also some studies achieved very higher (31.9%, 42.0%) [23, 35], a bit higher (20%) [7, 20, 22] and lower (8.6%, 3.7%) amount than that of our study result [21, 25]. In our study, the frequency of heterozygous and homozygous mutant genotypes were 36.6% and 5.2% in patients and 20.9% and 2.1% in controls, respectively. Our result is similar to Peng and et al study that in their study heterozygous and homozygous mutant genotypes was 41.0% and 7.2% in patients and 29.8 and 4.3 % in controls, respectively [7]. The results of some studies were consistent with the results of our study [7, 22, 36], and some were not [21, 23, 34, 37]. These differences in results could be due to genetic differences between populations, the number of people surveyed, single nucleotide polymorphisms interactions, genetic and environmental linkage, incorrect selection of the study groups, errors in study design, perform tests, and statistical analysis.

Our results identified the *CYP1A1* rs4646903 polymorphism as a significant risk factor for developing of early onset CAD. This finding signifies the importance of screening of this common genetic polymorphism in earlier age in high risk individuals. Guosheng Tu and et al reported that Lys198Asn polymorphism of endothelin-1 gene participate in early onset CAD [38]. In spite of critical role of *CYP1A1* rs4646903 polymorphism in developing of CAD especially in earlier ages, this genetic variation didn’t influence the severity of stenosis. Some other studies reported similar results [20] .Our study limitations were that we didn’t assay *CYP1A1* mRNA expression, enzymatic activity, ROS level, and their associations with mutant single nucleotide polymorphism due to financial and time constraints. We deduced that *CYP1A1* rs4646903 polymorphism is involved in the susceptibleness of people to CAD. However, this polymorphism didn’t involve in determining the severity of CAD. 

## Supplementary Materials

Supplement Fig. 1
