# Supplementary figures and images for: Association of the CYP1A1 rs4646903 polymorphism with susceptibility and severity of coronary artery disease
Source: Mol Biol Res Commun. 2021 Jun;10(2):22–61. doi: 10.22099/mbrc.2021.39141.1574 (PMC8310655; doi:10.22099/mbrc.2021.39141.1574)

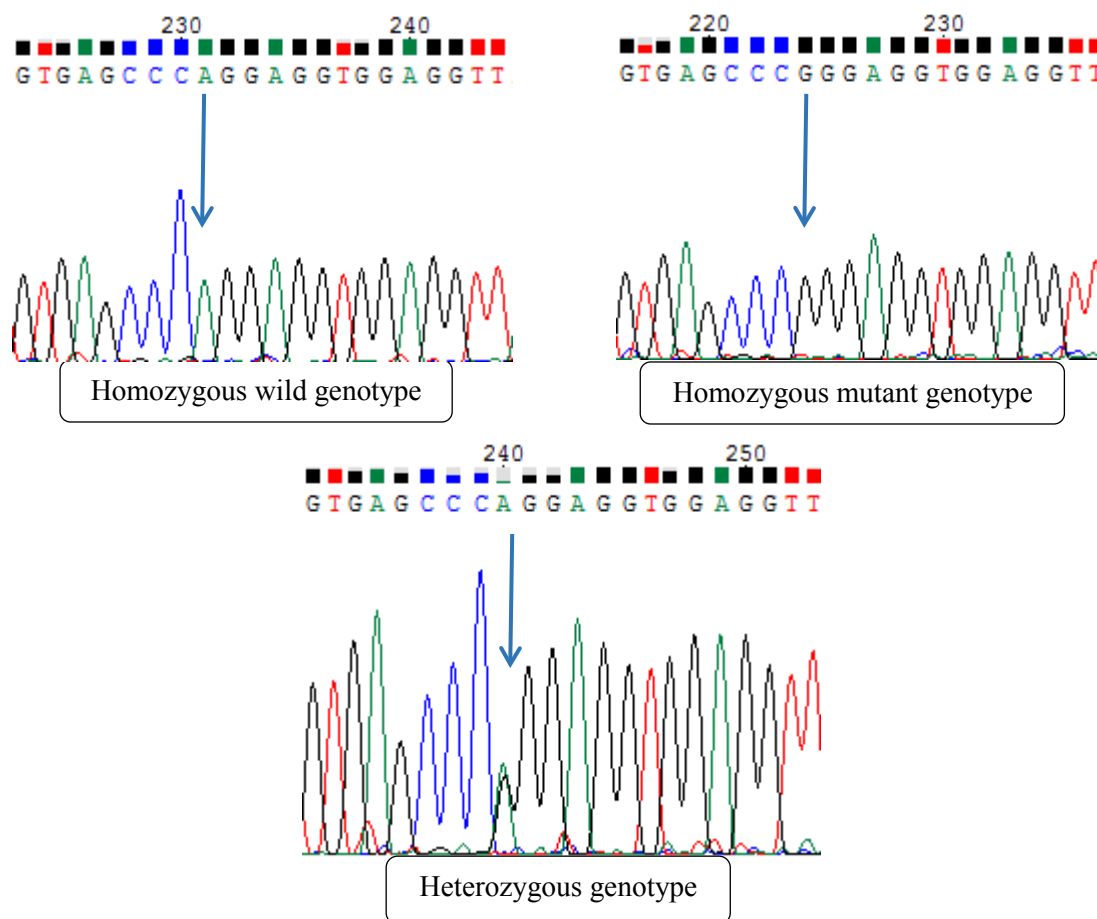

**Figure S1:** The result of *CYP1A1* rs4646903 sequencing.

Supplement: Supplement Fig. 1 [file mbrc-10-55-s001.pdf]
